# Evaluating COVID-19 vaccination intentions and vaccine hesitancy among parents of children with autism spectrum disorder

**DOI:** 10.1038/s41598-023-34191-y

**Published:** 2023-05-05

**Authors:** Mohamed Adil Shah Khoodoruth, Widaad Nuzhah Chut-kai Khoodoruth, Abd Alrhem Mohamad Ramadan, Beena Johnson, Shaima Gulistan, Raf Bernard Corvera Deluvio, Mohammed Nasser Alamri, Majid Al-Abdulla, Sami Ouanes, Yasser Saeed Khan

**Affiliations:** 1 Child and Adolescent Mental Health Service, Hamad Medical Corporation, PO Box 3050, Doha, Qatar; 2 Division of Genomics and Precision Medicine, College of Health and Life Sciences, Hamad Bin Khalifa University, Education City, Qatar; 3 Centre of Disease Control and Prevention Department, Hamad Medical Corporation, Doha, Qatar; 4 Child Development Center, Hamad Medical Corporation, Doha, Qatar; 5 Department of Pediatrics, Hamad Medical Corporation, Doha, Qatar; 6 Department of Psychiatry, Hamad Medical Corporation, Doha, Qatar; 7 College of Medicine, Qatar University, Doha, Qatar

**Keywords:** Infectious diseases, Neurological disorders, Psychiatric disorders, Risk factors, Disease prevention, Paediatrics, Public health

## Abstract

As the global vaccination mass campaign against COVID-19 extended to children aged 5 to 11 years, some parents remained hesitant about their children being administered the vaccine despite data supporting its safety. Parent vaccine hesitancy (PVH) may have predisposed certain groups of children, particularly those with autism spectrum disorder (ASD), to COVID-19 when other neurotypical children would have been vaccinated. We investigated the current PVH in 243 parents of children with ASD and 245 controls using the Parent Attitudes about Childhood Vaccines (PACV) scale. The study was conducted in Qatar from May to October 2022. Overall, 15.0% [95% CI 11.7%; 18.3%] of parents were vaccine-hesitant, with no difference (*p* = 0.054) between groups (ASD children [18.2%] vs. controls [11.7%]). The only sociodemographic factor associated with higher vaccine hesitancy was being a mother (as compared to being a father). The COVID-19 vaccine receipt rate at the time of the study did not differ between ASD (24.3%) and non-ASD groups (27.8%). Around two-thirds of parents of children with ASD refused or were unsure about vaccinating their children against COVID-19. We found that the intent to vaccinate against COVID-19 was higher in parents who were married and in those with a lower PACV total score. Continued public health efforts are needed to address vaccine hesitancy among parents.

## Introduction

Vaccination is considered one of the most important achievements of public health. Despite the life-saving benefits associated with prophylactic immunization of children and vaccine availability, there have been several outbreaks of potentially preventable diseases, such as measles and pertussis, internationally^1^. Vaccine hesitancy (VH) is a critical contributing factor, recognized by the World Health Organization (WHO) as a global health threat^2^. VH refers to a delay in acceptance or refusal of vaccines despite the availability of vaccine services, is complex and context-specific, varying across time, place, and vaccines, and is influenced by factors such as complacency, convenience, and confidence^3^. In fact, this definition depolarizes the pro- or anti-vaccine stance. VH is a complex and multi-layered phenomenon related to contextual influences, individual and group influences, and vaccine-specific issues^3^.

Vaccine hesitancy among parents of children with autism spectrum disorder (ASD) is recognized. ASD is a heterogenous, behaviorally defined neurodevelopmental disorder characterized by impairments in social interaction, communication, and restricted or repetitive behavioral patterns^4^. The prevalence of ASD has increased significantly in recent years, with the Center for Disease Control and Prevention (CDC, United States of America) now reporting estimates that 1 in 44 children are affected^5^. The historical controversy regarding the MMR vaccine (a vaccine against measles, mumps, and rubella given to young children) and ASD has rendered parents of children with ASD vulnerable to developing VH. Even though the possibility of any association between MMR and ASD has been persuasively disproven^6,7^, up to 40% of parents of children with ASD believe that vaccines contributed to or caused their child’s condition^8^. Furthermore, a child with ASD in a family can increase vaccine delay and refusal rates for that child and their younger siblings^9^. A study that compared parental vaccine hesitancy (PVH) among parents of children with ASD versus non-ASD developmental delay found that the former were significantly more likely to agree with “toxins in vaccines” as a cause of their child’s developmental delay^9^.

In the current era of information and the internet, traditional and social media play a crucial role in shaping individuals' decisions regarding vaccination^10,11^. Exposure to anti-vaccine websites or vaccine-critical blogs for as little as five to ten minutes can significantly alter an individual's perception and intent to vaccinate themselves and their loved ones^12,13^. Despite the logical fallacies, wishful thinking, and reality distortions on these websites^14^, the emotional impact of the stories and images of children purportedly harmed by vaccines can be profound, leaving a lasting impression on the unconscious mind and negatively influencing parental vaccine decision-making.

The Pfizer-BioNTech COVID-19 vaccine has been authorized for children aged 5 to 11 based on its safety, immunogenicity, and efficacy, amidst the ongoing COVID-19 pandemic that has resulted in over 762 million infections and 6.8 million deaths globally^15,16^. A diagnosis of ASD may increase the likelihood of developing anti-vaccine beliefs, but there is limited knowledge about PVH in this group during the COVID-19 pandemic and the rollout of mass vaccination campaigns for children.

As such, the objectives of this study were to (1) determine the prevalence of VH in parents/guardians of children with ASD in comparison with parents/guardians of neurotypical children, (2) investigate factors that may be associated with VH in parents of children with ASD, and (3) investigate the willingness of parents/guardians to vaccinate their children against COVID-19.

## Methods

### Study design and population

**Figure 1:**
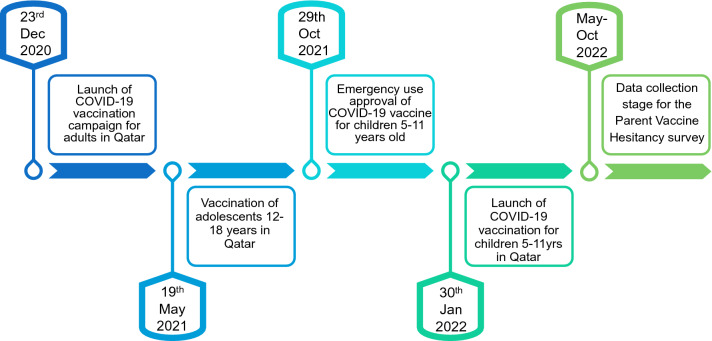
Vaccine administration development in Qatar.

This questionnaire-based, cross-sectional study was conducted after the start of the COVID-19 vaccination campaign for children 5–11 years, in Qatar, specifically from May to October 2022 (Fig. 1). Based on a 5% margin of error, 95% confidence interval, 1% prevalence of ASD^17^, and 20% response distribution. Our calculated sample size was 243 for each cohort.

Eligible parents/guardians received an invitation to participate by phone call. Based on their preference, they were emailed or texted a link to an online questionnaire (SurveyMonkey). Non-responsive families were notified up to three times about their opportunity to participate. 1170 parents were invited to participate in the study (n = 581 parents of children with ASD and n = 589 parents of neurotypical children). Of these, 488 parents (55.5% English-speaking and 44.5% Arabic-speaking) returned the completed questionnaire, with an overall response rate of 41.7%. There were no participant recruitment or compensation fees.

English- and Arabic-speaking parents or guardians of neurodiverse children 5–11 years of age with a primary diagnosis of ASD were eligible to participate in this cross-sectional assessment if their child received a formal diagnosis of ASD by the Child Development Center (5–8 years) and the Child and Adolescent Mental Health Service (8–11 years), both operating under the aegis of Hamad Medical Corporation, the state-funded major healthcare provider in Qatar.

English- and Arabic-speaking parents or guardians of neurotypical children 5–11 years of age without a neurodevelopmental or psychiatric disorder were recruited at the Pediatric Emergency Center (PEC), which provides clinical care to patients under 14 years of age and serves as a hub for other PECs across Qatar.

### Measures

#### The parent attitudes about childhood vaccines (PACV) survey

The PACV survey, a validated tool used in multiple countries, can successfully identify vaccine-hesitant parents and predict under-immunization in their children, particularly among those with high PACV scores. The PACV survey has also been previously applied to parents of children with ASD^8,9,18^. Both the English- and Arabic-PACV (validated) surveys were used in this study^19,20^. The PACV survey contains 15 items under three domains: behavior, safety and efficacy, and general attitude and trust (Supplementary Table S1). The PACV was scored by assigning a numeric score of 2 for non-demographic items answered with a hesitant response, a score of 1 for items answered with a response of “don’t know or not sure,” and a score of 0 for items answered with a non-hesitant response. PACV scores range from 0 to 100; a non-hesitant parent was defined with a score < 50 and a hesitant with a value ≥ 50. For converting the PACV raw score to the scale of 0–100, we used a simple linear transformation that accounts for missing data similar to that used by the original PACV author^19,21,22^.

#### The willingness of parents/guardians to vaccinate their children against COVID-19

We investigated the readiness of parents/guardians to vaccinate their children against COVID-19 by using three items [Has your child been vaccinated already with a dose of the COVID-19 vaccine?; Did you vaccinate your child with the COVID-19 vaccine mainly because it was mandatory for certain concessions (travel, entrance to places of leisure etc.)?; Do you intend to vaccinate your child with the COVID-19 vaccine?] rated as yes, no, not sure, or not applicable.

### Statistical analysis

#### Data was analyzed using IBM SPSS v26 for Windows

We determined absolute and relative frequencies for categorical variables. We calculated the mean and the standard deviation for normally distributed variables (as per Shapiro–Wilk’s test) and the median and the interquartile range (IQR) for non-normally distributed variables.

To examine associations between categorical variables, we used Pearson’s Chi-square and Fisher’s Exact Test in case of non-validity. We compared means using t-test for independent variables (when the number of means was two) and one-way analysis of variance (ANOVA) when the number of means was three or more.

We calculated the PACV score in accordance with the original PACV system^19,20^. For items 3 and 4, “don’t know” responses were considered as missing since they reflected a problem with rather than vaccine hesitancy.

To compare PACV total scores between parents of ASD children and parents of neurotypical children, we conducted an analysis of covariance (ANCOVA) with the PACV total score as a dependent variable, the diagnostic group (ASD vs. control) as a fixed factor, and child’s sex, child’s age, being a first child, responder’s relationship to the child, parent’s citizenship status, parent’s educational level, and parent’s marital status as covariates. Homogeneity of variances was verified using Levene’s test. Effect size was estimated using partial Eta squared (η^2^).

To assess the relationship between the intent to vaccinate one’s child against COVID-19 and PACV score, we built a binary logistic regression model with the intention to vaccinate (item 20 dichotomized as “1” for “Yes” and “0” for “No” or “Not sure”) as the dependent variable, and the following variables as independent variables: PACV total score, group (ASD vs. controls), child’s sex, child’s age, being a first child, responder’s relationship to the child, parent’s citizenship status, parent’s educational level, and parent’s marital status. All variables were entered using the “ENTER” method with no specific order. We calculated the odds ratio and its 95% confidence interval. We checked for multicollinearity by calculating the Variance Inflation Factor (VIF). The VIF scores were less than 10, indicating that multicollinearity was not present in the data.

For multiple, comparisons, we used the Holm-Bonferroni’s method to adjust the p values.

For all analyses, we used an alpha value of 0.05.

### Ethical considerations

This study was reviewed and approved by the institutional review board at Hamad Medical Corporation (MRC-01-22-126). All methods were performed in accordance with the relevant guidelines and regulations of the Medical Research Center of Hamad Medical Corporation. Participation was voluntary. Phone calls were made to all parents/guardians included in the sample to invite them to the study. All information relevant to the study, including its purpose, impact on clinical care, and confidentiality safeguards, were provided. This was done using an invitation form with a standard script developed specifically for this study in both English and Arabic. Informed consent was obtained from participating parents/legal guardians. Responses were not tagged with any personally identifiable information. All data was anonymized after collection.

## Results

We included 243 parents with ASD and 245 parents of control (neurotypical children) in the study. Results of Pearson's chi-square test revealed that children with ASD were more likely to be male (77.4%, n = 181 vs. 55%, n = 133, *p* < 0.001) and that they parents were more likely to be Qatari (8.6%, n = 21 vs. 2.9%, n = 7, *p* = 0.006) than their neurotypical counterparts. Results of the t-test for independent groups showed that children with ASD were significantly younger than their neurotypical counterparts (7.4 ± 1.8 years vs. 8 ± 1.8, *p* < 0.001). Other sociodemographic characteristics did not differ between parents of patients with ASD and parents of controls (Table 1).

**Table 1:** Sociodemographic characteristics of parents of patients with autism spectrum disorder and parents of controls.

	Parents of children with ASD, n (%)	Parents of controls, n (%)	p*	Effect size measure**
n	243	245		
Age of the child, years (m ± SD)	7.4 ± 1.8	8 ± 1.8	< 0.001	0.348
Sex of the child, male	181 (77.4)	133 (55.0)	< 0.001	0.236
First child	114 (46.9)	132 (53.9)	0.124	0.070
Relationship to the child				
Mother	127 (52.3)	112 (45.7)	0.240	0.076
Father	115 (47.3)	115 (46.9)		
Other	1 (0.4)	3 (1.2)		
Parent’s age group				
18–29 years old	7 (2.9)	5 (2.0)	0.549	0.027
30 years or older	236 (97.1)	240 (98.0)		
Parent’s marital status				
Single	1 (0.4)	0 (0.0)	0.425	
Married	234 (96.3)	234 (95.5)		
Widowed	1 (0.4)	4 (1.6)		
Divorced	7 (2.9)	7 (2.9)		
Parent’s education, higher education	206 (84.8)	208 (84.9)	0.969	0.063
Number of children in the household				
1	28 (11.4)	35 (14.4)	0.199	0.098
2	72 (29.4)	87 (35.8)		
3	78 (31.8)	61 (25.1)		
4 or more	67 (27.3)	60 (24.7)		
Citizenship status				
Qatari National	21 (8.6)	7 (2.9)	0.022	0.125
Qatari resident	186 (76.5)	202 (82.4)		
Other	36 (14.8)	36 (14.7)		

Since the results of this table were exploratory, no correction for multiple comparisons was made.

*ASD* autism spectrum disorder, *COVID-19* coronavirus disease 2019.

*t-test for independent groups was used to compare age of the child. For all other variables, Pearson’s Chi-square was used.

**Cohen’s d was used for age of the child, Cramer-V was used for all other variables.

Around one-quarter of children had been vaccinated against COVID-19 at the time of the study, with no difference between groups. In both groups, about one-third of the parents stated that the main reason for this vaccination was that it was mandatory for certain concessions (travel, entrance to leisure places, etc.).

Most (78.4%) of caregivers of children with ASD agree/strongly agree with the item ‘I trust the information I receive about shots’, and 84.5% agreed/strongly agreed that they could openly discuss their concerns about shots with their child's doctor. The answers to the PACV questionnaire are detailed in Supplementary Table S1.

Cronbach's alpha for the PACV scale in our study was 0.636, which is deemed as fair.

The overall prevalence of vaccine hesitancy was 15.0% [95% CI 11.7%; 18.3%] (defined by a total PACV score > 50); 18.2% (n = 42) [95% CI 13.2%; 23.2%] in the ASD group vs. 11.7% (n = 42) [95% CI 7.5%; 16.0%] in the control group. The difference was not statistically significant (*p* = 0.054). The PACV total score did not significantly differ between parents of children with ASD and parents of controls (Table 2).

**Table 2:** COVID-19 vaccination in children with ASD and controls and vaccine hesitancy in their parents.

	Parents of children with ASD, n (%)	Parents of controls, n (%)	p*	Effect size measure**
Has your child been vaccinated already with a dose of the COVID-19 vaccine?	59 (24.3)	68 (27.8)	1.000	0.040
Did you vaccinate your child with the COVID-19 vaccine mainly because it was mandatory for certain concessions (travel, entrance to places of leisure etc.)?	39 (35.5)	37 (30.8)	1.000	0.049
Do you intend to vaccinate your child with the COVID-19 vaccine?				
Yes	71 (35.7)	79 (42.0)	1.000	0.065
No	85 (42.7)	73 (38.8)		
Not sure	43 (21.6)	36 (19.1)		
PACV total score (m ± SD)	40.8 ± 9.6	42.4 ± 9.8	0.328	0.165

*ASD* autism spectrum disorder, *COVID-19* coronavirus disease 2019, *PACV* parent attitudes about childhood vaccines.

*t-test for independent groups was used to compare PACV total score. For all other variables, Pearson’s Chi-square was used.

**Cohen’s d was used for PACV total score, Cramer-V was used for all other variables.

p values were adjusted for multiple comparison using Holm-Bonferroni’s method.

Even after controlling for covariates (child’s sex, child’s age, being a first child, responder’s relationship to the child, parent’s citizenship status, parent’s educational level, and parent’s marital status), ANCOVA analysis did not show any difference in PACV score between parents of children with ASD and parents of controls (*F* = 1.769, *p* = 0.184, *η*^2^ = 0.004). Among covariates, only the relationship to the child was associated with PACV score, where mothers were significantly more skeptical than fathers (*F* = 24.551, *p* < 0.001, *η*^2^ = 0.054) (Table 3).

**Table 3:** Analysis of covariance (ANCOVA) comparing parent attitudes about childhood vaccines scores between parents of patients with autism spectrum disorder and parents of controls.

Variable	Type III sum of squares	df	Mean square	F	Sig	Partial eta squared
Tests of between-subjects effects						
Corrected model	3901.955^a^	8	487.744	5.705	< 0.001	0.096
Intercept	14,360.258	1	14,360.258	167.959	< 0.001	0.280
Child’s gender	32.475	1	32.475	0.380	0.538	0.001
Child’s age	44.596	1	44.596	0.522	0.471	0.001
Parent’s citizenship (Qatari vs non-Qatari)	306.130	1	306.130	3.581	0.059	0.008
Parent’s marital status (married vs non-married)	270.113	1	270.113	3.159	0.076	0.007
Parent’s educational level (higher education vs no higher education)	269.207	1	269.207	3.149	0.077	0.007
First child	161.501	1	161.501	1.889	0.170	0.004
Relationship to the child	2099.088	1	2099.088	24.551	< 0.001	0.054
Group (parents with ASD children vs controls)	151.250	1	151.250	1.769	0.184	0.004

^a^R squared = .096 (Adjusted R squared = .079).

When we examined the variables associated with the intent to vaccinate one’s child against COVID-19, logistic binary regression analysis had a Nagelkerke R^2^ = 0.169. The analysis revealed that the intent to vaccinate was higher in parents who were married (*p* = 0.018, OR = 2.324 [1.155; 4.674]) versus those who were not married, and in those with a lower PACV total score (*p* < 0.001; OR = 0.940 [0.913; 0.969]). In other terms, parents who were married and who had a lower vaccine hesitancy were more likely to intend to vaccinate their children against COVD-19. Other variables, including group (ASD vs. controls), child’s sex, child’s age, being a first child, responder’s relationship to the child, parent’s citizenship status, and parent’s educational level, were not found to be associated with the intent to vaccinate the child against COVID-19 (Table 4).

**Table 4:** Variables associated with the intent to vaccinate one’s child against COVID-19—Logistic binary regression with the intent to vaccinate as the dependent variable.

	Wald	p	OR	95% CI for OR		VIF
				Lower	Upper	
Child’s gender	0.409	0.522	1.181	0.709	1.967	1.063
Child’s age	1.196	0.274	1.073	0.946	1.218	1.047
First Child	1.010	0.315	0.789	0.497	1.252	1.015
Relationship to the child	0.164	0.686	1.103	0.687	1.769	1.114
Parent’s citizenship (Qatari vs non-Qatari)	3.612	0.057	0.135	0.017	1.065	1.076
Parent’s marital status (married vs non-married)	5.594	0.018	2.324	1.155	4.674	1.060
Parent’s educational level (Higher Education vs no higher education)	2.846	0.092	3.828	0.805	18.203	1.025
PACV total score	16.451	< 0.001	0.940	0.913	0.969	1.100
Group (parents with ASD children vs controls)	0.084	0.772	0.932	0.578	1.502	1.097
Constant	0.090	0.764	0.683			

*ASD* autism spectrum disorder, *COVID-19* coronavirus disease 2019, *PACV* parent attitudes about childhood vaccines, *VIF* variance inflation factor.

## Discussion

To the best of our knowledge, this study is the first to directly compare vaccine hesitancy among parents of children with ASD to parents of neurotypical children in the COVID-19 era right after the launch of COVID-19 vaccination in children 5–11 years old and to examine parent-reported COVID-19 vaccination intent to vaccinate their child. In this study, 18.2% of parents of children with ASD were vaccine-hesitant, based on the PACV score, which was lower than vaccine hesitancy previously noted among parents of children with ASD in recent studies^8,9,18^. Possible reasons for the reassuring level of PVH in Qatar could be the encouraging levels of trust in the information parents received about vaccines and the ability to openly discuss with their child’s doctor as physician trust and governmental distrust play significant roles in PVH^23^. Our study had a nearly equal number of mothers and fathers participating. Mothers have been reported to be more concerned and skeptical than fathers in previous studies^24^, a finding confirmed by our study. This could be attributed to mothers adopting a cautious approach regarding the potential adverse effects of vaccines, even if the scientific evidence supporting their concerns is uncertain. Moreover, vaccine-hesitant mothers have reported a preference toward natural immunity, and some of them were more distressed with the idea of injecting an “artificial product” into the “pure bodies” of children^25^.

The rate of PVH in our ASD sample (18.2%) was greater than that reported in other studies reporting on neurotypical children, using the same tool form: Turkey (13.8%)^26^, United Arab Emirates (12%)^20^, Malaysia (11.6%)^27^ and Iraq (9.9%)^28^. This could result from parental concern about potential vaccine side effects and safety, as almost one in two parents were “somewhat” and “very concerned” about these concerns. Furthermore, some parents probably still believe in the possible link between vaccines and autism because of the previously reported association between autism and the MMR vaccine in the infamous Wakefield article, published in The Lancet in 1998 but retracted in 2010 because of fabricated data^29,30^. In our study, however, we did not find that parents of ASD children had a significantly higher rate of VH than controls. This may mean that this alleged association between vaccines and ASD either did not have any significant impact on our participants or had the same effect on parents regardless of whether they had a child with ASD.

When assessing the parents’ intentions to vaccinate their children with ASD against COVID-19, we found that only about a third intended to vaccinate their children. One potential reason for vaccine hesitancy among parents may be the belief that children are at lower risk of contracting and experiencing severe illness from COVID-19, despite evidence suggesting that young children with underlying medical conditions and very young age are still vulnerable^31,32^. Additionally, the media coverage surrounding two healthcare workers in the UK who experienced anaphylactic reactions to the Pfizer/BioNTech vaccine may have contributed to concerns about vaccine allergies and contraindications^33^. The prevalence of misinformation about vaccines, particularly for newer vaccines, has been identified as the primary cause of vaccine hesitancy, as evidence shows how misinformation about vaccines led to lower vaccine intentions and uptakes^34^. Parents and caregivers will take any suggestion that vaccines are a possible cause of a dreaded disease or syndrome seriously, and the natural tendency of risk aversion might set in.

The level of PVH, specific to the COVID-19 vaccine, in the ASD arm described here was similar to that described in an American^35^ and a Japanese study^36^ that explored PVH in children with ASD and attention-deficit hyperactivity disorder (ADHD), respectively. As for two-thirds of parents who refuse or are undecided about vaccinating their children against COVID-19, healthcare providers should reinforce the benefits of vaccines and provide education and evidence-based recommendations to parents who hold erroneous vaccine beliefs about risks, benefits and current evidence, especially those related to autism. Such strategies targeting vaccine beliefs may improve the likelihood of COVID-19 vaccination among children with autism and prevent continued disparities in COVID-19 outcomes for this population, given that individuals with ASD and other comorbidities have a higher likelihood of hospitalizations and elevated length of hospital stay from COVID-19 infections^37^. Further investigation is necessary to examine how procedural pain and fear impact children with autism, given that their cognitive pathways for pain processing may be compromised^38^. In fact, the pain and fear experienced by children during vaccinations can significantly impact parents' vaccination experiences and adherence rates, with negative vaccination experiences resulting in an increased risk of under-vaccination for infants^39^.

One of the study’s main strengths was that we surveyed a representative sample of parents and guardians of children with ASD in Qatar during the COVID-19 pandemic right after the launch of COVID-19 vaccination in children 5–11 years old. We also recruited caregivers of children without ASD for comparison. Another robust point is that COVID-19 vaccines are freely available to residents and citizens of Qatar, eliminating factors such as vaccine availability, supply, and costs that could impact PVH. Limitations of the study were the self-report characteristic of parent vaccine intentions, which may be subject to social desirability bias. There was an under-representation of Qatari Nationals in the control group. However, it is unlikely this would significantly impact the study’s results. This is because Qatari Nationals are well-represented in the ASD arm (exposure of interest). Moreover, we had no information regarding the non-respondents and could not determine the differences between the respondents and the non-respondents. Some of the results (mainly the comparison of sociodemographic variables between groups) were exploratory, and therefore no correction for multiple comparisons was made for these particular findings. Lastly, COVID-19 vaccination status was based on parental reports and was not validated with medical records.

## Conclusion

This study suggests that there may not be differences in vaccine hesitancy in parents of children with ASD compared with parents of neurotypical children. We found an overall PVH rate of 15.0%. We also found that VH was higher in mothers. Around two-thirds of parents of children with ASD refused or were unsure about vaccinating their children against COVID-19. However, the similar rate of prior COVID-19 vaccine receipt among ASD children and their counterparts was an assuring observation. Continued public health efforts are needed to address vaccine hesitancy among parents. This can reduce setbacks in the care of children with neurodevelopmental disabilities. More qualitative studies are required in order better to understand the causes and expressions of autism-related PVH. More research is also needed on the impact of the degree of fear and needles among children with ASD on their parents and caregivers.

## Supplementary Information

Supplementary Information.

## Data Availability

All data generated or analyzed during this study are included in this published article (and its supplementary information file).

## References

[1] Phadke VK, Bednarczyk RA, Salmon DA, Omer SB (2016). Association between vaccine refusal and vaccine-preventable diseases in the United States: A review of Measles and Pertussis. JAMA.

[2] Centers for Disease Control and Prevention (CDC). Ten great public health achievements--worldwide, 2001–2010. *MMWR. Morb. Mortal. Wkly. Rep.* **60**, 814–8 (2011). 21697806

[3] MacDonald NE (2015). Vaccine hesitancy: Definition, scope and determinants. Vaccine.

[4] American Psychiatric Association (2022). Neurodevelopmental disorders. Diagn. Stat. Man. Ment. Disord..

[5] Centers for Disease Control and Prevention (CDC). Data & Statistics on Autism Spectrum Disorder. https://www.cdc.gov/ncbddd/autism/data.html (2015).

[6] Kalkbrenner AE, Schmidt RJ, Penlesky AC (2014). Environmental chemical exposures and autism spectrum disorders: A review of the epidemiological evidence. Curr. Probl. Pediatr. Adolesc. Health Care.

[7] Taylor LE, Swerdfeger AL, Eslick GD (2014). Vaccines are not associated with autism: An evidence-based meta-analysis of case-control and cohort studies. Vaccine.

[8] Sahni LC (2020). Vaccine hesitancy and illness perceptions: comparing parents of children with autism spectrum disorder to other parent groups. Child. Heal. Care.

[9] Bonsu NEM (2021). Understanding vaccine hesitancy among parents of children with autism spectrum disorder and parents of children with non-autism developmental delays. J. Child Neurol..

[10] Scullard P, Peacock C, Davies P (2010). Googling children’s health: Reliability of medical advice on the internet. Arch. Dis. Child..

[11] Galdi, S., Arcuri, L. & Gawronski, B. Automatic mental associations predict future choices of undecided decision-makers. *Science (80-. ).* **321**, 1100–1102 (2008). 10.1126/science.1160769 18719288

[12] Nan X, Madden K (2012). HPV vaccine information in the blogosphere: How positive and negative blogs influence vaccine-related risk perceptions, attitudes, and behavioral intentions. Health Commun..

[13] Betsch C, Renkewitz F, Betsch T, Ulshöfer C (2010). The influence of vaccine-critical websites on perceiving vaccination risks. J. Health Psychol..

[14] Jacobson RM, Targonski PV, Poland GA (2007). A taxonomy of reasoning flaws in the anti-vaccine movement. Vaccine.

[15] World Health Organization. Coronavirus disease (COVID-19) dashboard. https://covid19.who.int/ (2021). 37184163

[16] Walter EB (2021). Evaluation of the BNT162b2 covid-19 vaccine in children 5–11 years of age. N. Engl. J. Med..

[17] Alshaban F (2019). Prevalence and correlates of autism spectrum disorder in Qatar: A national study. J. Child Psychol. Psychiatry Allied Discip..

[18] Goin-Kochel RP (2020). Beliefs about causes of autism and vaccine hesitancy among parents of children with autism spectrum disorder. Vaccine.

[19] Opel DJ (2011). Development of a survey to identify vaccine-hesitant parents: The parent attitudes about childhood vaccines survey. Hum. Vaccin..

[20] Alsuwaidi AR (2020). Vaccine hesitancy and its determinants among Arab parents: A cross-sectional survey in the United Arab Emirates. Hum. Vaccines Immunother..

[21] Opel DJ (2011). Validity and reliability of a survey to identify vaccine-hesitant parents. Vaccine.

[22] Opel DJ (2013). The relationship between parent attitudes about childhood vaccines survey scores and future child immunization status: A validation study. JAMA Pediatr..

[23] Rozbroj T, Lyons A, Lucke J (2020). Vaccine-hesitant and vaccine-refusing parents’ reflections on the way parenthood changed their attitudes to vaccination. J. Community Health.

[24] Suran M (2022). Why parents still hesitate to vaccinate their children against COVID-19. JAMA.

[25] Dubé E (2016). Nature does things well, why should we interfere?. Qual. Health Res..

[26] Yörük S, Güler D (2021). Factors associated with pediatric vaccine hesitancy of parents: A cross-sectional study in Turkey. Hum. Vaccines Immunother..

[27] Mohd Azizi, F. S., Kew, Y. & Moy, F. M. Vaccine hesitancy among parents in a multi-ethnic country, Malaysia. *Vaccine* **35**, 2955–2961 (2017). 10.1016/j.vaccine.2017.04.010 28434687

[28] Raof, A. M. Parental attitude and beliefs towards child vaccination: identifying vaccine hesitant groups in a family health center, Erbil City, Iraq. *World Fam. Med. Journal/Middle East J. Fam. Med.* **16**, 17–26 (2018).

[29] Eggertson, L. Lancet retracts 12-year-old article linking autism to MMR vaccines. *CMAJ : Can. Med. Assoc. J. ***182**, E199–E200 (2010). 10.1503/cmaj.109-3179 PMC2831678 20142376

[30] DeStefano F, Shimabukuro TT (2019). The MMR vaccine and autism. Annu. Rev. Virol..

[31] Hoang A (2020). COVID-19 in 7780 pediatric patients: A systematic review. E Clin. Med..

[32] Tsabouri S, Makis A, Kosmeri C, Siomou E (2021). Risk factors for severity in children with Coronavirus Disease 2019: A comprehensive literature review. Pediatr. Clin. N. Am..

[33] Glover RE, Urquhart R, Lukawska J, Blumenthal KG (2021). Vaccinating against covid-19 in people who report allergies. The BMJ.

[34] Ngai CSB, Singh RG, Yao L (2022). Impact of COVID-19 vaccine misinformation on social media virality: Content analysis of message themes and writing strategies. J. Med. Internet Res..

[35] Choi K, Becerra-Culqui T, Bhakta B, Bruxvoort K, Coleman KJ (2022). Parent intentions to vaccinate children with autism spectrum disorder against COVID-19. J. Pediatr. Nurs..

[36] Tsai CS, Hsiao RC, Chen YM, Yen CF (2021). Factors related to caregiver intentions to vaccinate their children with attention-deficit/hyperactivity disorder against covid-19 in Taiwan. Vaccines.

[37] Karpur A, Vasudevan V, Shih A, Frazier T (2022). Brief Report: Impact of COVID-19 in individuals with autism spectrum disorders: Analysis of a national private claims insurance database. J. Autism Dev. Disord..

[38] Yasuda Y (2016). Sensory cognitive abnormalities of pain in autism spectrum disorder: A case-control study. Ann. Gen. Psychiatry.

[39] Stockwell MS, Irigoyen M, Martinez RA, Findley S (2011). How parents’ negative experiences at immunization visits affect child immunization status in a community in New York City. Public Health Rep..

