# Supplementary material for: Evaluating COVID-19 vaccination intentions and vaccine hesitancy among parents of children with autism spectrum disorder
Source: Sci Rep. 2023 May 5;13:7353. doi: 10.1038/s41598-023-34191-y (PMC10161974; doi:10.1038/s41598-023-34191-y)
Supplement: Supplementary file 1 — Supplementary Information. [file 41598_2023_34191_MOESM1_ESM.docx]

**Supplementary Table 1: Descriptive statistics – Answers to the different items of the Parent Attitudes About Childhood Vaccines questionnaire**

|  | |  |  |
| --- | --- | --- | --- |
|  |  |  |  |
|  |  | **Parents of children with ASD** | **Parents of controls** |
| **Have you ever delayed having your child get a shot (not including seasonal flu or swine flu (H1N1) shots) for reasons other than illness or allergy?** | Yes | 26(10.6%) | 37(15.2%) |
|  | No | 203(82.9%) | 198(81.5%) |
|  | Don't know | 16(6.5%) | 8(3.3%) |
| **Have you ever decided not to have your child get a shot (not including seasonal flu or swine flu (H1N1) shots) for reasons other than illness or allergy?** | Yes | 19(7.8%) | 19(7.8%) |
|  | No | 215(87.8%) | 218(89.7%) |
|  | Don't know | 11(4.5%) | 6(2.5%) |
| **How sure are you that following the recommended shot schedule is a good idea for your child?** | 0 (Not at all sure) | 15(6.1%) | 17(7.0%) |
|  | 1 | 1(0.4%) | 3(1.2%) |
|  | 2 | 1(0.4%) | 5(2.1%) |
|  | 3 | 0(0.0%) | 4(1.6%) |
|  | 4 | 1(0.4%) | 1(0.4%) |
|  | 5 | 27(11.0%) | 16(6.6%) |
|  | 6 | 11(4.5%) | 7(2.9%) |
|  | 7 | 21(8.6%) | 16(6.6%) |
|  | 8 | 25(10.2%) | 27(11.1%) |
|  | 9 | 25(10.2%) | 36(14.8%) |
|  | 10 (Completely sure) | 118(48.2%) | 111(45.7%) |
| **Children get more shots than are good for them** | Strongly Agree | 24(9.8%) | 20(8.2%) |
|  | Agree | 59(24.1%) | 67(27.6%) |
|  | Not Sure | 93(38.0%) | 76(31.3%) |
|  | Disagree | 49(20.0%) | 56(23.0%) |
|  | Strongly Disagree | 20(8.2%) | 24(9.9%) |
| **I believe that many of the illnesses that shots prevent are severe** | Strongly Agree | 52(21.2%) | 54(22.2%) |
|  | Agree | 135(55.1%) | 129(53.1%) |
|  | Not Sure | 45(18.4%) | 44(18.1%) |
|  | Disagree | 10(4.1%) | 13(5.3%) |
|  | Strongly Disagree | 3(1.2%) | 3(1.2%) |
| **It is better for my child to develop immunity by getting sick than to get a shot** | Strongly Agree | 22(9.0%) | 32(13.2%) |
|  | Agree | 57(23.3%) | 47(19.3%) |
|  | Not Sure | 68(27.8%) | 41(16.9%) |
|  | Disagree | 76(31.0%) | 94(38.7%) |
|  | Strongly Disagree | 22(9.0%) | 29(11.9%) |
| **It is better for children to get fewer vaccines at the same time** | Strongly Agree | 19(7.8%) | 29(11.9%) |
|  | Agree | 82(33.5%) | 92(37.9%) |
|  | Not Sure | 88(35.9%) | 65(26.7%) |
|  | Disagree | 48(19.6%) | 47(19.3%) |
|  | Strongly Disagree | 8(3.3%) | 10(4.1%) |
| **How concerned are you that your child might have a serious side effect from a shot?** | Not at all concerned | 6(2.4%) | 17(7.0%) |
|  | Not too concerned | 44(18.0%) | 32(13.2%) |
|  | Not Sure | 48(19.6%) | 34(14.0%) |
|  | Somewhat concerned | 81(33.1%) | 81(33.3%) |
|  | Very concerned | 66(26.9%) | 79(32.5%) |
| **How concerned are you that anyone of the childhood shots might not be safe?** | Not at all concerned | 15(6.1%) | 21(8.6%) |
|  | Not too concerned | 53(21.6%) | 43(17.7%) |
|  | Not Sure | 54(22.0%) | 48(19.8%) |
|  | Somewhat concerned | 74(30.2%) | 71(29.2%) |
|  | Very concerned | 49(20.0%) | 60(24.7%) |
| **How concerned are you that a shot might not prevent the disease?** | Not at all concerned | 16(6.5%) | 23(9.5%) |
|  | Not too concerned | 60(24.5%) | 60(24.7%) |
|  | Not Sure | 71(29.0%) | 60(24.7%) |
|  | Somewhat concerned | 61(24.9%) | 66(27.2%) |
|  | Very concerned | 37(15.1%) | 34(14.0%) |
| **If you had another infant today, would you want him/her to get all the recommended shots?** | Yes | 204(83.3%) | 201(82.7%) |
|  | No | 18(7.3%) | 18(7.4%) |
|  | Don't know | 23(9.4%) | 24(9.9%) |
| **Overall, how hesitant about childhood shots would you consider yourself to be?** | Not at all Hesitant | 77(31.4%) | 59(24.3%) |
|  | Not too Hesitant | 88(35.9%) | 91(37.4%) |
|  | Not Sure | 28(11.4%) | 23(9.5%) |
|  | Somewhat Hesitant | 38(15.5%) | 51(21.0%) |
|  | Very Hesitant | 14(5.7%) | 19(7.8%) |
| **I trust the information I receive about shots** | Strongly Agree | 63(25.7%) | 52(21.4%) |
|  | Agree | 129(52.7%) | 123(50.6%) |
|  | Not Sure | 41(16.7%) | 47(19.3%) |
|  | Disagree | 8(3.3%) | 10(4.1%) |
|  | Strongly Disagree | 4(1.6%) | 11(4.5%) |
| **I am able to openly discuss my concerns about shots with my child's doctor** | Strongly Agree | 68(27.8%) | 63(25.9%) |
|  | Agree | 139(56.7%) | 128(52.7%) |
|  | Not Sure | 27(11.0%) | 38(15.6%) |
|  | Disagree | 10(4.1%) | 9(3.7%) |
|  | Strongly Disagree | 1(0.4%) | 5(2.1%) |
| **All things considered, how much do you trust your child’s doctor?** | 0 (Do not trust at all) | 5(2.0%) | 6(2.5%) |
|  | 1 | 0(0.0%) | 1(0.4%) |
|  | 2 | 2(0.8%) | 4(1.6%) |
|  | 3 | 4(1.6%) | 7(2.9%) |
|  | 4 | 1(0.4%) | 6(2.5%) |
|  | 5 | 28(11.4%) | 18(7.4%) |
|  | 7 | 53(21.6%) | 41(16.9%) |
|  | 8 | 44(18.0%) | 48(19.8%) |
|  | 9 | 41(16.7%) | 45(18.5%) |
|  | 10 (Completely Trust) | 67(27.3%) | 67(27.6%) |
